# Clinical manifestations of schizophrenia in four patients with variants in voltage‐gated calcium channel‐encoding genes: a case series

**DOI:** 10.1111/pcn.13494

**Published:** 2022-11-09

**Authors:** Tzuyao Lo, Itaru Kushima, Branko Aleksic, Akira Yoshimi, Toshiyuki Someya, Yuichiro Watanabe, Norio Ozaki

**Affiliations:** ^1^ Department of Psychiatry Nagoya University Graduate School of Medicine Nagoya Japan; ^2^ Medical Genomics Center Nagoya University Hospital Nagoya Japan; ^3^ Division of Clinical Sciences and Neuropsychopharmacology Faculty and Graduate School of Pharmacy, Meijo University Nagoya Japan; ^4^ Department of Psychiatry Niigata University Graduate School of Medical and Dental Sciences; ^5^ Institute for Glyco‐core Research Nagoya University Nagoya Japan

Voltage‐gated calcium channels (VGCCs) play an important role in synaptic functions and are implicated in the pathogenesis of schizophrenia (SCZ).^1^ Rare or *de novo* genetic variants in VGCC genes have been shown to be associated with SCZ and other psychiatric disorders.^2^, ^3^, ^4^, ^5^ However, clinical manifestations in psychiatric patients with such variants have not been well described. Therefore, we present clinical data from four Japanese patients with SCZ and rare or *de novo* variants (copy number variants [CNVs] and a missense variant) affecting three VGCC genes (*CACNA1C*, *CACNA1H*, and *CACNA2D1*) identified in our previous studies.^6^, ^7^, ^8^ The genes encode pore‐forming and auxiliary subunits of VGCCs and were indicated as SCZ‐associated genes (Supplementary M5, M6).^4^, ^5^ The present study was approved by the ethics committee of Nagoya University and Niigata University. Written informed consent was obtained from all participants. Detailed methods are described in the Supplementary materials Appendix S1.

The clinical data of the four patients with SCZ are summarized in Fig. 1a.

**Fig. 1:**
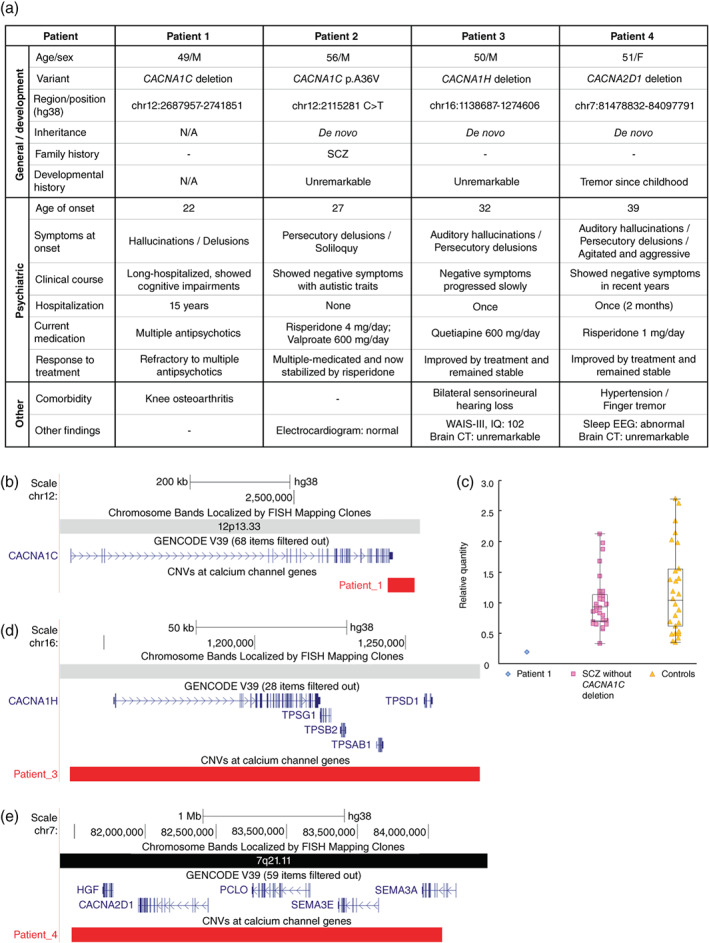
Clinical data, CNVs of VGCC genes, and gene expression results. (a) Summary of the clinical data from four patients with SCZ with variants in VGCC genes. (b) The 54‐kb deletion in patient 1 spans exons 46–47 of the *CACNA1C* gene (transcript NM_000719.6), corresponding to the C‐terminal end of CACNA1C. (c) Reduced mRNA expression level of *CACNA1C* in patient 1. The box plot represents the distribution of gene expression levels of *CACNA1C* mRNA; each dot represents the relative expression of samples including patient 1, 29 patients with SCZ without *CACNA1C* deletion, and 28 healthy controls, calculated using the 2‐ΔΔCt method. (Supplementary M3). (d) The 136‐kb deletion in patient 3 spans the entire *CACNA1H* gene. (e) The 2.6‐Mb deletion in patient 4 spans the entire *CACNA2D1* gene. CNVs, copy number variants; CT, computed tomography; EEG, electroencephalogram; IQ, intelligence quotient; N/A, not available; SCZ, schizophrenia; VGCC, Voltage‐gated calcium channel; WAIS‐III, Wechsler Adult Intelligence Scale‐III.

Patient 1 was a 49‐year‐old male with rare *CACNA1C* deletion (Fig. 1b). Before onset, he worked for 3 years. He developed delusions and hallucinations at age 22 years. His psychotic symptoms were refractory to multiple antipsychotics. He was hospitalized for 15 years, during which time, his functioning was significantly reduced. At the time of this study, he had severe cognitive impairment and was being treated with multiple antipsychotics. Gene expression analysis revealed a reduced level of *CACNA1C* mRNA expression (Fig. 1c).

Patient 2 was a 56‐year‐old male with a *de novo* missense variant (p.A36V) in *CACNA1C* that has a gain‐of‐function effect on CACNA1C.^8^ He had a family history of SCZ (elder sister). After graduating from university, he worked for 3 years. Delusional mood occurred at age 23 years, and persecutory delusions and soliloquy developed at age 27 years. He was treated with multiple antipsychotics, but later switched to risperidone. Positive symptoms had been unremarkable in the past 15 years, and he mainly manifested negative symptoms such as low motivation and poor hygiene. He showed autistic traits such as insistence on sameness. At the time of this study, his symptoms were being controlled by risperidone (4 mg/day) and valproate (600 mg/day), the latter of which was prescribed for irritability.

Patient 3 was a 50‐year‐old male with *de novo CACNA1H* deletion (Fig. 1d). At age 32 years, he developed auditory hallucinations and persecutory delusions. Psychotic symptoms were improved with treatment, but negative symptoms progressed slowly. At the time of this study, he was stabilized with quetiapine (600 mg/day). His intelligence quotient score (Wechsler Adult Intelligence Scale‐III) was 102, and brain computed tomography (CT) revealed no significant findings. He had bilateral sensorineural hearing loss.

Patient 4 was a 51‐year‐old female with *de novo CACNA2D1* deletion (Fig. 1e). She had a history of finger tremor since childhood. At age 39 years, she developed persecutory delusions and auditory hallucinations and became agitated and aggressive. After being hospitalized, these symptoms were immediately improved by antipsychotics. After discharge, her condition remained stable. At the time of this study, she was being treated with risperidone (1 mg/day). She mainly manifested negative symptoms, including social withdrawal and apathy. Brain CT revealed no significant findings. She showed abnormal electroencephalogram patterns during sleep involving slow‐wave burst‐firing lasting several seconds, predominantly over the bilateral frontal and central regions.

Here, we provide the first report of clinical manifestations of SCZ in patients with variants in VGCC genes. The *de novo* or rare variants affected SCZ‐associated genes, supporting their pathogenicity. *CACNA1C* deletion, which reduces gene expression and has a loss‐of‐function effect, was identified in patient 1 with severe cognitive impairment and treatment resistance. Interestingly, similar phenotypes have been reported in individuals carrying larger deletions involving *CACNA1C* and other genes (Supplementary D1). Mice with conditional‐knockout of *Cacna1c* in forebrain glutamatergic neurons have exhibited cognitive impairments and other SCZ‐related behavioral abnormalities.^9^ Therefore, dysfunction in glutamatergic synapses may underlie the cognitive impairments and psychoses in patient 1. Conversely, *CACNA1C* p.A36V, which has a gain‐of‐function effect on VGCC and impairs neuronal Ca^2+^ homeostasis,^8^ was identified in patient 2, along with autistic traits. Gain‐of‐function variants in *CACNA1C* can lead to Timothy syndrome, which is characterized by autism (Supplementary D2). Patients 3 and 4 had neurological traits associated with VGCC dysfunction, including tremor and hearing loss.^10^ However, the deletions they carry also affect genes other than VGCC genes (Supplementary D3). Identification of *de novo* VGCC genetic variants supports a role for VGCC genes in the pathophysiology of SCZ while various clinical manifestations suggest phenotypic heterogeneity.

## Disclosure statement

T.L., B.A., A.Y., T.S. and Y.W. declare no conflicts of interest. I.K. has received a research grant from the SENSHIN Medical Research Foundation. N.O. has received research support or speakers' honoraria from, or has served as a consultant to, Sumitomo Dainippon, Eisai, Otsuka, KAITEKI, Mitsubishi Tanabe, Shionogi, Eli Lilly, Mochida, DAIICHI SANKYO, Nihon Medi‐Physics, Takeda, Meiji Seika Pharma, EA Pharma, Pfizer, MSD, Lundbeck Japan, Taisho Pharma, Viatris, Kyowa Kirin, and TSUMURA outside the submitted work.

## Supporting information


**Appendix S1.** Supplementary Materials. Supplementary Methods and Supplementary Discussions

## References

[1] Nanou E , Catterall WA . Calcium channels, synaptic plasticity, and neuropsychiatric disease. Neuron 2018; 98: 466–481. 29723500 10.1016/j.neuron.2018.03.017

[2] Szatkiewicz JP , Fromer M , Nonneman RJ *et al*. Characterization of single gene copy number variants in schizophrenia. Biol. Psychiatry 2020; 87: 736–744. 31767120 10.1016/j.biopsych.2019.09.023 PMC7103483

[3] Nakatochi M , Kushima I , Ozaki N . Implications of germline copy‐number variations in psychiatric disorders: Review of large‐scale genetic studies. J. Hum. Genet. 2021; 66: 25–37. 32958875 10.1038/s10038-020-00838-1

[4] Singh T , Poterba T , Curtis D *et al*. Rare coding variants in ten genes confer substantial risk for schizophrenia. Nature 2022; 604: 509–516. 35396579 10.1038/s41586-022-04556-w PMC9805802

[5] Purcell SM , Moran JL , Fromer M *et al*. A polygenic burden of rare disruptive mutations in schizophrenia. Nature 2014; 506: 185–190. 24463508 10.1038/nature12975 PMC4136494

[6] Kushima I , Aleksic B , Nakatochi M *et al*. Comparative analyses of copy‐number variation in autism Spectrum disorder and schizophrenia reveal etiological overlap and biological insights. Cell Rep. 2018; 24: 2838–2856. 30208311 10.1016/j.celrep.2018.08.022

[7] Kushima I , Aleksic B , Nakatochi M *et al*. High‐resolution copy number variation analysis of schizophrenia in Japan. Mol. Psychiatry 2017; 22: 430–440. 27240532 10.1038/mp.2016.88

[8] Wang C , Horigane SI , Wakamori M *et al*. Identification of ultra‐rare disruptive variants in voltage‐gated calcium channel‐encoding genes in Japanese samples of schizophrenia and autism spectrum disorder. Transl. Psychiatry 2022; 12: 84. 35220405 10.1038/s41398-022-01851-y PMC8882172

[9] Dedic N , Pöhlmann ML , Richter JS *et al*. Cross‐disorder risk gene CACNA1C differentially modulates susceptibility to psychiatric disorders during development and adulthood. Mol. Psychiatry 2018; 23: 533–543. 28696432 10.1038/mp.2017.133 PMC5822460

[10] Zamponi GW . Targeting voltage‐gated calcium channels in neurological and psychiatric diseases. Nat. Rev. Drug Discov. 2016; 15: 19–34. 26542451 10.1038/nrd.2015.5

