# Supplementary material for: Clinical manifestations of schizophrenia in four patients with variants in voltage‐gated calcium channel‐encoding genes: a case series
Source: Psychiatry Clin Neurosci. 2022 Nov 9;77(1):57–9. doi: 10.1111/pcn.13494 (PMC10099977; doi:10.1111/pcn.13494)
Supplement: Supplementary file 1 — Appendix S1. Supplementary Materials. Supplementary Methods and Supplementary Discussions [file PCN-77-57-s001.docx]

Supplementary materials

**Clinical manifestations of schizophrenia in four patients with variants in voltage-gated calcium channel-encoding genes: a case series**

Running title: VGCC gene variants in schizophrenia

Tzuyao Lo^1,†^, MD, Itaru Kushima^1,2,†*^, MD, PhD, Branko Aleksic^1^, MD, PhD, Akira Yoshimi^3^, PhD, Toshiyuki Someya^4^, MD, PhD, Yuichiro Watanabe^4^, MD, PhD and Norio Ozaki^1,5^, MD, PhD

^1^ Department of Psychiatry, Nagoya University Graduate School of Medicine, Nagoya, Japan

^2^ Medical Genomics Center, Nagoya University Hospital, Nagoya, Japan

^3^ Division of Clinical Sciences and Neuropsychopharmacology, Faculty and Graduate School of Pharmacy, Meijo University, Nagoya, Japan

^4^ Department of Psychiatry, Niigata University Graduate School of Medical and Dental Sciences

^5^ Institute for Glyco-core Research, Nagoya University, Nagoya, Japan

†These authors contributed equally to this work.

*Corresponding author:

Itaru Kushima, MD, PhD

Department of Psychiatry, Nagoya University Graduate School of Medicine

65 Tsurumai-Cho, Showa-ku, Nagoya, Aichi 466-8550, Japan

Phone: +81 52 7442282; Fax: +81 52 7442293

E-mail: [kushima@med.nagoya-u.ac.jp](mailto:kushima@med.nagoya-u.ac.jp)

Supplementary Methods

**M1. Participants**

All patients were of Japanese ancestry and were diagnosed according to the Diagnostic and Statistical Manual of Mental Disorders, Fifth Edition, criteria for schizophrenia (SCZ). This study was approved by the ethics committee of Nagoya University Graduate School of Medicine, Niigata University and other participating institutes. Written informed consent was obtained from all patients.

**M2. Genetic analysis**

Genomic DNA was extracted from blood samples. Copy number variants (CNVs) in voltage-gated calcium channel (VGCC) genes were identified in three patients (Patients 1, 3, and 4) using array comparative genomic hybridization (NimbleGen 720k Whole-Genome Tiling Arrays; Roche NimbleGen, Madison, WI) ^1, 2^. We generated CNV calls by using Nexus Copy Number software, v9.0 (BioDiscovery, El Segundo, CA). The Ion PGM sequencing platform (Thermo Fisher Scientific, Waltham, MA, USA) was utilized to identify a *de novo* missense variant (p.A36V) of *CACNA1C* in Patient 2 ^3^. The variant was validated by Sanger sequencing. All genomic locations are given in hg38 coordinates.

**M3. Gene expression analysis**

Lymphoblastoid cell lines (LCLs) were established according to widely used Epstein–Barr virus transformation with minor modification. These LCLs were derived from Patient 1 with *CACNA1C* deletion, patients with SCZ without *CACNA1C* deletion (n=29), and healthy controls (n=28). Total RNA was extracted from LCLs using the RNAqueous Kit (Ambion, Austin, TX) and treated with DNase to remove contaminated genomic DNA using the TURBO DNA-free Kit (Ambion), and then reverse-transcribed to cDNA using the High Capacity RNA-to-cDNA Kit (Applied Biosystems, Foster City, CA). Two housekeeping genes, beta-2-microglobulin (*B2M*) and glucuronidase-beta (*GUSB*), were selected as internal control genes to normalize the polymerase chain reaction (PCR). Quantitative PCR was performed on an ABI prism 7900HT Real-Time PCR System (Applied Biosystems) using predesigned TaqMan Gene Expression Assay probes (Hs00930485_m1 for *CACNA1C*, Hs99999907_m1 for *B2M*, and Hs99999908_m1 for *GUSB*; Applied Biosystems). Measurement of the cycle threshold was performed in duplicate. The data, including amplifying efficiency and relative expression on quantification, were analyzed using the comparative cycle threshold method.

**M4. Phenotypic analysis**

We retrospectively collected clinical data from the medical records of four patients with SCZ with variants in VGCC-encoding genes. The data evaluated included developmental history, family history, medical history, psychiatric symptoms, age at onset of SCZ, history of hospitalizations, medications, treatment response or resistance, and other clinical manifestations.

**M5. The function of VGCC genes**

Three VGCC genes (*CACNA1C*, *CACNA1H*, and *CACNA2D1*) were included in this case series. VGCCs consist of different subunits, including the pore-forming α1 subunit, as well as the auxiliary α2δ and β subunits^4^. The properties of VGCCs (e.g., kinetics, voltage dependence, etc.) are principally determined by the α1 subunits, while many of the properties could also be modulated by the β and α2δ auxiliary subunits. *CACNA1C* encodes the pore-forming subunit α1C in L-type Ca_V_1.2 channels, the predominant calcium channel in the ventricular cardiac muscle which is also present throughout the brain^5^. *CACNA1H* encodes the pore-forming subunit α1H in T-type Ca_V_3.2 channels, the key regulators of neuronal excitability in the peripheral and central nervous systems which contribute to low-voltage evoked exocytosis^6^. *CACNA2D1* encodes the auxiliary subunit α2δ1 in Ca_V_1 and Ca_V_2 channels, which is expressed in neurons, axonal terminals, and dendrites throughout the central and peripheral nervous system^7^. The auxiliary α2δ subunits also have major roles in trafficking the Ca_V_1 and Ca_V_2 channels to the plasma membrane and specific domains of polarized cells, including neurons^5^.

**M6. Schizophrenia Exome Sequencing Meta-analysis (SCHEMA) consortium**

SCHEMA consortium is a large multi-site collaboration dedicated to aggregating, generating, and analyzing high-throughput sequencing data of SCZ patients to improve the understanding of disease architecture and advance gene discovery ^8^. The Phase I data set contains the analysis of exomes from 24,248 SCZ cases and 97,322 controls, and *de novo* mutations from 3,402 parent-proband trios. Taking advantage of these data, we were able to examine whether damaging variants in specific genes were overrepresented in patients with SCZ. Damaging variants (Class I variants) were defined as loss-of-function variants (stop-gained, frameshift, and essential splice donor or acceptor variants) and missense variants with MPC pathogenicity score >3 ^9^. Damaging variants were jointly analyzed in a single case-control burden test. Case-control significance was evaluated using a permutation-based Fisher's Exact Test. We examined whether damaging variants in a specific VGCC gene included in the current study (*CACNA1C*, *CACNA1H*, and *CACNA2D1*) were overrepresented in patients with SCZ. As a result, overrepresentation of Class I variants was observed in *CACNA1C* (odds ratio=6.02, p=0.0185) and *CACNA2D1* (odds ratio=4.59, p=0.000680), indicating their role in the pathogenesis of SCZ, while the result was insignificant in *CACNA1H* (odds ratio=1.45, p=0.404). The detailed data on the overrepresentation of Class I variants in *CACNA1C*, *CACNA1H*, and *CACNA2D1* are provided below. Additionally, in a large whole exome sequencing study of SCZ, multiple rare disruptive variants in VGCC genes including *CACNA1C*, *CACNA1H*, and *CACNA2D1* were identified in SCZ, promoting VGCC as one of the most enriched gene sets with rare disruptive variants in SCZ^5, 10^.

| Gene name | Cases | Controls | Odds Ratio | Case/Control P-value |
| --- | --- | --- | --- | --- |
| *CACNA1C* | 6 | 4 | 6.02 | 0.0185 |
| *CACNA1H* | 13 | 36 | 1.45 | 0.404 |
| *CACNA2D1* | 8 | 7 | 4.59 | 0.000680 |

Supplementary Discussions

**D1. Previous reports of deletions involving *CACNA1C***

To our knowledge, there were no previous reports of patients with SCZ with *CACNA1C* deletion. However, there were two reports of individuals with psychotic manifestations with larger deletions at 12p13, which involve *CACNA1C* and other genes ^11, 12^. Interestingly, individuals in both reports exhibited cognitive disability, while in one report the psychotic symptoms were also difficult to control with psychotropic medications ^11^. Of note, the deletions in these two reports (4.5-Mb and 6.2-Mb) were much larger than the deletion in our study (54-kb) and they involved many other genes (at least 18 genes), while the deletion in our report involved solely *CACNA1C*.

**D2. Gain-of-function variant in patient 2**

The missense variant (p.A36V) in *CACNA1C* in patient 2 was found to cause a significant reduction in Ca^2+^-dependent inactivation (CDI), a negative feedback mechanism limiting excessive Ca^2+^ entry, thereby increasing Ca^2+^ influx^3^. This gain-of-function effect on neuronal Ca_V_1.2 calcium channels may lead to an impairment of neuronal Ca^2+^ homeostasis, which is hypothesized to be involved in the pathogenesis of SCZ^13^. Additionally, the gain-of-function variant in *CACNA1C* has been identified as the cause of Timothy syndrome, a channelopathy characterized by autism. The underlying mechanism was considered to be related to gain-of-function changes to Ca_V_1.2 channel gating, including reduced inactivation of Ca_V_1.2 calcium channels, which may lead to increased Ca^2+^ influx^14^. We therefore suspect the gain-of-function variant in patient 2 may be related to autistic traits.

**D3. Deletions in patient 1, 3 and 4**

Patient 1 carries a 54-kb deletion which spans exons 46–47 of the *CACNA1C* gene (transcript NM_000719.6). Patient 3 carries a 136-kb deletion which spans the entire *CACNA1H* gene. Patient 4 carries a 2.6-Mb deletion which spans the entire *CACNA2D1* gene. However, the deletions they carry may affect genes other than VGCC genes (referred to as “non-VGCC genes” in the followings), namely *ITFG2-AS1* and *LINC02371* in patient 1, *TPSG1*, *TPSB2*, *TPSAB1*, and *TPSD1* in patient 3, and *HGF*, *PCLO*, *SEMA3E*, and *SEMA3A* in patient 4. While non-VGCC genes in patient 1 and 3 were not reported to be associated with psychiatric or neurological disorders, there were reports indicating a role for non-VGCC genes in patient 4 in psychiatric disorders or neural development, including *PCLO^15^*, *SEMA3E^16^*, and *SEMA3A^17^*. Overrepresentation of damaging variants in *PCLO* in patients with SCZ was identified in SCHEMA^8^ (odds ratio = 4.02; p-value = 0.000130)*.* Notably, previous studies reported increased expression of *PCLO* in the prefrontal cortex of bipolar disorder patients^15^ and increased expression of *SEMA3A* in the cerebellum in SCZ patients^17^, which was contrary to the effect of deletion in our report. Data from the SCHEMA consortium have shown overrepresentation of damaging variants in *CACNA1C* and *CACNA2D1*, supporting their role in the pathogenesis of SCZ^8^. Additionally, there was evidence suggesting neurological traits in patient 3 and patient 4 (hearing loss and tremor) may be related to calcium channel dysfunction. A recent case report reported a Saudi female with a heterozygous variant in the *CACNA1H* who had epilepsy and hearing loss ^18^. Another case report identified a null *CACNA2D2* variant that abolished α2δ2 subunit (isoform of α2δ1 encoded by *CACNA2D1*) expression in a patient with neurological traits including tremor^19^. Overall, although we cannot rule out the possibility that psychiatric and neurological traits in patient 1, 3 and 4 may be associated with non-VGCC genes, there is evidence supporting their association with VGCC genes. Still, the fact that deletions in patient 1, 3 and 4 affect genes other than VGCC genes should be considered as one limitation of this study.

**References**

1. Kushima I, Aleksic B, Nakatochi M, et al. Comparative Analyses of Copy-Number Variation in Autism Spectrum Disorder and Schizophrenia Reveal Etiological Overlap and Biological Insights. Cell Rep 2018;24:2838-2856.

2. Kushima I, Aleksic B, Nakatochi M, et al. High-resolution copy number variation analysis of schizophrenia in Japan. Mol Psychiatry 2017;22:430-440.

3. Wang C, Horigane SI, Wakamori M, et al. Identification of ultra-rare disruptive variants in voltage-gated calcium channel-encoding genes in Japanese samples of schizophrenia and autism spectrum disorder. Transl Psychiatry 2022;12:84.

4. Catterall WA. Voltage-gated calcium channels. Cold Spring Harb Perspect Biol 2011;3:a003947.

5. Heyes S, Pratt WS, Rees E, et al. Genetic disruption of voltage-gated calcium channels in psychiatric and neurological disorders. Prog Neurobiol 2015;134:36-54.

6. Cai S, Gomez K, Moutal A, Khanna R. Targeting T-type/CaV3.2 channels for chronic pain. Transl Res 2021;234:20-30.

7. Cui W, Wu H, Yu X, Song T, Xu X, Xu F. The Calcium Channel α2δ1 Subunit: Interactional Targets in Primary Sensory Neurons and Role in Neuropathic Pain. Front Cell Neurosci 2021;15:699731.

8. Singh T, Poterba T, Curtis D, et al. Rare coding variants in ten genes confer substantial risk for schizophrenia. Nature 2022;604:509-516.

9. Samocha KE, Kosmicki JA, Karczewski KJ, et al. Regional missense constraint improves variant deleteriousness prediction. BioRxiv 2017:148353.

10. Purcell SM, Moran JL, Fromer M, et al. A polygenic burden of rare disruptive mutations in schizophrenia. Nature 2014;506:185-190.

11. Velinov M, Beldia G, Gu H, Tsiouris JA, Jenkins EC, Brown WT. Psychotic manifestations in a patient with mental retardation and a 6.2 megabase deletion at the distal short arm of chromosome 12. CNS Spectr 2008;13:515-519.

12. Vargas H, Beldia G, Korosh W, et al. A 4.5 Mb terminal deletion of chromosome 12p helps further define a psychosis-associated locus. Eur J Med Genet 2012;55:573-576.

13. Bojarski L, Debowska K, Wojda U. In vitro findings of alterations in intracellular calcium homeostasis in schizophrenia. Prog Neuropsychopharmacol Biol Psychiatry 2010;34:1367-1374.

14. Marcantoni A, Calorio C, Hidisoglu E, Chiantia G, Carbone E. Cav1.2 channelopathies causing autism: new hallmarks on Timothy syndrome. Pflugers Arch 2020;472:775-789.

15. Choi KH, Higgs BW, Wendland JR, Song J, McMahon FJ, Webster MJ. Gene expression and genetic variation data implicate PCLO in bipolar disorder. Biol Psychiatry 2011;69:353-359.

16. Deloulme JC, Gory-Fauré S, Mauconduit F, et al. Microtubule-associated protein 6 mediates neuronal connectivity through Semaphorin 3E-dependent signalling for axonal growth. Nat Commun 2015;6:7246.

17. Eastwood SL, Law AJ, Everall IP, Harrison PJ. The axonal chemorepellant semaphorin 3A is increased in the cerebellum in schizophrenia and may contribute to its synaptic pathology. Mol Psychiatry 2003;8:148-155.

18. Algahtani HA, Shirah BH, Samman A, Alhazmi A. Epilepsy and Hearing Loss in a Patient with a Rare Heterozygous Variant in the. J Epilepsy Res 2022;12:33-35.

19. Pippucci T, Parmeggiani A, Palombo F, et al. A novel null homozygous mutation confirms CACNA2D2 as a gene mutated in epileptic encephalopathy. PLoS One 2013;8:e82154.
